# Glial Contribution to Excitatory and Inhibitory Synapse Loss in Neurodegeneration

**DOI:** 10.3389/fncel.2019.00063

**Published:** 2019-02-26

**Authors:** Christopher M. Henstridge, Makis Tzioras, Rosa C. Paolicelli

**Affiliations:** ^1^Centre for Discovery Brain Sciences, The University of Edinburgh, Edinburgh, United Kingdom; ^2^Dementia Research Institute UK, The University of Edinburgh, Edinburgh, United Kingdom; ^3^Department of Physiology, University of Lausanne, Lausanne, Switzerland

**Keywords:** microglia, astrocytes, neurodegeneration, synapse loss, E/I imbalance

## Abstract

Synapse loss is an early feature shared by many neurodegenerative diseases, and it represents the major correlate of cognitive impairment. Recent studies reveal that microglia and astrocytes play a major role in synapse elimination, contributing to network dysfunction associated with neurodegeneration. Excitatory and inhibitory activity can be affected by glia-mediated synapse loss, resulting in imbalanced synaptic transmission and subsequent synaptic dysfunction. Here, we review the recent literature on the contribution of glia to excitatory/inhibitory imbalance, in the context of the most common neurodegenerative disorders. A better understanding of the mechanisms underlying pathological synapse loss will be instrumental to design targeted therapeutic interventions, taking in account the emerging roles of microglia and astrocytes in synapse remodeling.

## Introduction

The prevalence of neurodegenerative disorders has been rapidly increasing over the past decades. These untreatable and often lethal conditions including Alzheimer’s disease (AD), Parkinson’s disease (PD), amyotrophic lateral sclerosis (ALS) and multiple sclerosis (MS), affect over 100 million people worldwide (Prince et al., 2013; Browne et al., 2014; Baxter et al., 2015). Despite differences in age of onset and genetic risk factors associated with the disease, common pathophysiological features can be identified, including synaptic and glial dysfunction, as well as cognitive impairments. Synapse loss is an early occurring hallmark in many neurodegenerative disorders, which correlates best with the appearance and progression of cognitive decline (DeKosky and Scheff, 1990; Terry et al., 1991; Koffie et al., 2011; Spires-Jones and Hyman, 2014). Abnormal glial function is also recognized as an early pathological feature commonly observed in neurodegenerative disease (Verkhratsky et al., 2014). However, for a long time, the prevailing neuro-centric view of pathogenesis has led to the underestimation of key roles for non-neuronal cells in the brain, primarily glia, which were instead considered as mere bystanders or secondary responders in the pathological process. Only in the last decade, with the advent of new genetic, molecular and pharmacological tools, have significant steps forward been made towards our understanding of glial function, revealing a central role for these cells in disease (Verkhratsky et al., 2014).

From the Greek word “glue,” glia in the central nervous system (CNS) include three major cell subsets: astrocytes, microglia and oligodendrocytes. While the latter are mainly responsible for the formation of myelin and for providing metabolic support to axons (Baumann and Pham-Dinh, 2001; Simons and Nave, 2015), microglia and astrocytes cover a variety of functions, ranging from trophic support to refinement and coordination of neural networks (Reemst et al., 2016; Allen and Lyons, 2018). Microglia are the resident macrophages of the CNS and constitute about 10%–15% of all the brain cells. Historically, they have been regarded exclusively as innate immune cells, considered to be “activated” only during infection or injury. In the last decade, however, several new physiological roles for microglia have been described, revealing a much broader scenario for the multifaceted tasks performed by these cells (Tremblay et al., 2011; Sierra et al., 2014; Paolicelli and Ferretti, 2017). Astrocytes are the more abundant glial cell in the CNS; their processes closely enwrap synapses, and their role in regulating synaptogenesis, neurotransmitter recycling and synaptic transmission is well established (Parpura et al., 1994; Vesce et al., 1999; Panatier et al., 2011; Chever et al., 2016). In addition, they play key roles in maintaining the blood–brain barrier, providing trophic and metabolic support to neurons (Pellerin et al., 2007; Sofroniew and Vinters, 2010). Since the recent recognition of a role for glia in refining synaptic connections, intense investigations have been devoted to elucidate the molecular mechanisms of glia-meditated synapse elimination, particularly in the context of neurodegeneration. The majority of neurodegenerative disorders fall into the category of “proteinopathies,” because of the characteristic accumulation of toxic protein aggregates (Ross and Poirier, 2004; Soto and Pritzkow, 2018). In such diseases, pathological proteins often accumulate at the synapse (Koffie et al., 2009, 2012; Henstridge et al., 2018), thus causing synaptic dysfunction and likely rendering the synapses vulnerable and primed for removal (Walsh et al., 2002; Geracitano et al., 2003; Pieri et al., 2003; Shankar et al., 2007; Crimins et al., 2012). In the case of AD, for instance, amyloid β (Aβ) peptide accumulates at the synaptic site long before its extracellular aggregation in plaques, and it is associated with alterations in synaptic structures, both in mouse and in human studies (Gylys et al., 2004; Almeida et al., 2005; Sokolow et al., 2012; Takahashi et al., 2013).

Synapse elimination could occur *via* autonomous pathways within the damaged neuron, due to localized caspases or necrotic signals (Wishart et al., 2006; Ertürk et al., 2014) or *via* active non-cell-autonomous removal of synapses by surrounding glial cells (Hong et al., 2016; Vasek et al., 2016; Paolicelli et al., 2017). Evidence for either scenario or even a combination of both exists. In this review article, we will primarily focus on glial-dependent synapse loss and revise the recent literature providing evidence for glial contribution to excitatory-inhibitory network dysfunction in pathological states.

## Synapse Remodeling in Development and Disease

The term synapse, from the Greek σνυάψις, meaning “conjunction,” refers to the physical point of contact between two neurons, and thus defines the anatomical site of information exchange between an axonal input and the recipient dendritic spine (Harris and Weinberg, 2012). Synapses are highly dynamic sub-cellular structures, as they can be rapidly formed or eliminated during plasticity-mediated processes (Engert and Bonhoeffer, 1999; Matsuzaki et al., 2001). They represent the structural basis of long-term potentiation (LTP), essential for memory formation (Matsuzaki et al., 2004). Evidence of the highly dynamic nature of synapses has been provided by advances in live imaging techniques, showing that dendritic spines rapidly appear and disappear as a result of experience-dependent plasticity upon sensory experience, and learning processes (Toni et al., 1999; Lendvai et al., 2000; Holtmaat and Svoboda, 2009; Fu et al., 2012). During early development, immature neural circuits undergo synaptic refinement, in which activity-dependent competition between synapses ultimately results in the elimination of inappropriate connections and brain plasticity, while strong synapses are reinforced (Penn et al., 1998; Lichtman and Colman, 2000; Hua and Smith, 2004; Torborg and Feller, 2005; Mikuni et al., 2013; Fields et al., 2014; Robin et al., 2018). Importantly, the proposed mechanism of the strongest “winning inputs” (Personius and Balice-Gordon, 2000) is consistent across a number of models, namely the neuromuscular junction (NMJ; Wang et al., 2014), the Purkinje fibers in the cerebellum (Mason and Gregory, 1984; Hashimoto and Kano, 2003; Kakegawa et al., 2015) and the retino-thalamic system (Hong and Chen, 2011), suggesting that activity-dependent remodeling of synapses is a conserved process across the central and peripheral nervous system. *In vivo* imaging studies recently showed that monocular deprivation (MD) increases dendritic spine elimination in the developing mouse visual cortex, with no effects on synapse formation (Zhou et al., 2017). Interestingly, binocular deprivation (BD), which entirely suppresses competition between the two eyes, failed to induce synapse elimination, and resulted by contrast in enlarged dendritic spine size (Zhou et al., 2017).

The high dynamic remodeling of synapses not only occurs during early developmental stages, but also persists across the entire lifespan (Peretti et al., 2015). Live imaging of cortical regions largely supports the experience-dependent plasticity of dendritic spines in the adult mouse brain (Xu et al., 2009; Yang and Zhou, 2009). *In vivo* imaging of the hippocampus, a highly plastic structure, has been made possible only recently, upon novel methods of cortical tissue resection (Pilz et al., 2016). Such studies have provided evidence for network plasticity with new spines formed and eliminated in the CA1 *stratum radiatum*, with an impressive-previously underscored- spine turnover of ~40% within 4 days (Gu et al., 2014; Pfeiffer et al., 2018).

While synapse elimination in the context of brain development and experience-dependent plasticity is a physiological process (Wolff and Missler, 1993; Kamiyama et al., 2006), its later and dysregulated occurrence is recognized as an early pathological feature of neurodegenerative diseases (DeKosky and Scheff, 1990; Henstridge et al., 2018). Indeed, one of the earliest hallmarks of neurodegeneration is the loss of presynaptic terminals and dendritic spines, which represents the major correlate of cognitive impairment (Terry et al., 1991; Scheff et al., 2006, 2014). Structural and functional alterations of synapses, culminating in synapse loss, are associated with sensory, motor, and cognitive impairments observed in a variety of neurodegenerative disorders, ranging from AD to motor neuron diseases (MND), and often precede clinical manifestations (Selkoe, 2002; Henstridge et al., 2018). Yet, the causes and the molecular mechanisms leading to pathological synapse loss have not been fully elucidated (Henstridge et al., 2016). On one hand, the regenerative capacity of synapses seems to be significantly reduced in the disease state, as shown in prion-infected and AD mouse models (Peretti et al., 2015); on the other hand, aberrant synaptic pruning or lack of trophic support by surrounding glia cells can contribute to the drastic reduction in synapse number.

## Contribution of Glia to Synapse Elimination

**Figure 1:**
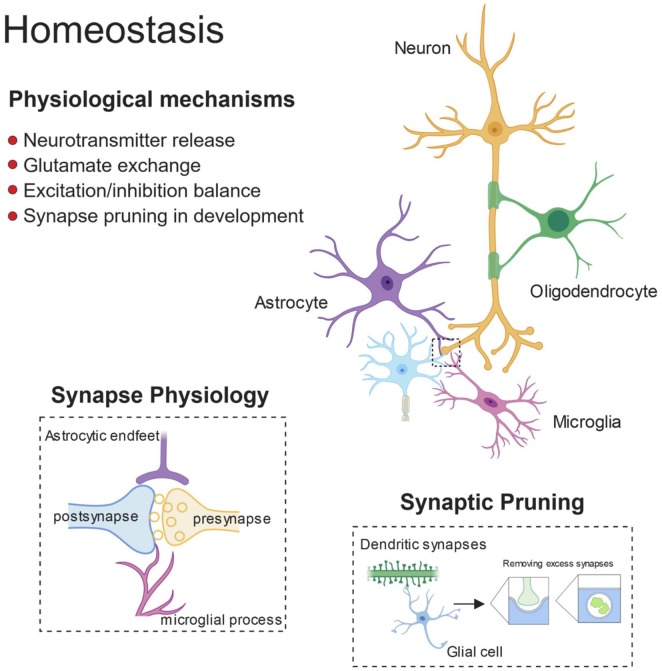
Glial control of synaptic homoeostasis. Synapses exists as tri- or even quad-partite structures with glial processes in direct contact with neuronal components. Glia play important roles in regulating efficient neurotransmitter release and clearance, as well as providing trophic factors to ensure healthy function. Furthermore, during development glia prune away excess synapses and by doing so, fine-tune the excitatory/inhibitory balance within developing neuronal networks.

Recent literature highlights glial cells as active participants in the process of neural circuits refinement (Figure 1). Microglia and astrocytes contribute to accurate network formation by directly pruning redundant synapses during early development, and thus shaping brain connectivity (Paolicelli et al., 2011; Chung et al., 2013; Hakim et al., 2014; Zhan et al., 2014; Risher et al., 2014; Filipello et al., 2018). In addition, glia can also act indirectly to induce effects on synaptic function, *via* the release of soluble modulators (Chung et al., 2015). Compelling evidence shows that synapse elimination by glia is important in the activity-dependent wiring of the brain, with microglia and astrocytes selectively removing the weaker synapses upon input competition (Schafer et al., 2012; Chung et al., 2013; Sipe et al., 2016; Yang et al., 2016). For example, the visual system is a well-characterized model for experience-dependent synaptic refinement (Wiesel and Hubel, 1963), and thus, the developing retino-thalamic system has been frequently used for studying competition of synaptic inputs, which project from the retinal ganglion cells (RGCs) to the relay neurons in the dorsal later geniculate nucleus (dLGN), and then to the primary visual cortex. This model has been influential in revealing that microglia are active players in experience-dependent remodeling of neural circuits (Tremblay et al., 2010; Schafer et al., 2012; Sipe et al., 2016). Sensory deprivation, by closure of one eye, during the visual critical period results in enhanced engulfment of synaptic terminals by microglia and astrocytes (Chung et al., 2013; Sipe et al., 2016), whereas BD, achieved by pharmacological blockade, drastically reduces astrocyte-mediated synaptic pruning, further confirming that active competition of synaptic inputs is required for glial-dependent synapse remodeling (Chung et al., 2013). Several pathways have been implicated in this process, including fractalkine signaling (Cx3cr1/Cx3cl1), DAP12/triggering receptor expressed on myeloid cells 2 (TREM2) signaling and the complement pathway for synapse elimination by microglia (Paolicelli et al., 2011; Schafer et al., 2012; Filipello et al., 2018), and MEGF10 and MERTK for astrocyte-mediated synapse engulfment (Chung et al., 2013). Dysfunctional regulation of such pathways or intrinsic defects in glia are possible causes for the pathological synaptic pruning observed in neurodegeneration. Indeed, a growing body of evidence indicates that glia-mediated synapse removal becomes dysregulated in aging and disease. A prominent hypothesis is that an increased activation of the complement cascade, associated with neurodegenerative disorders, enhances complement deposition on synaptic terminals, priming the synapses for removal and thus mediating aberrant synapse elimination. In support of this, distinct animal models of neurodegeneration (discussed below) exhibit upregulated levels of complement C3 and C1q, and subsequent synapse loss (Fonseca et al., 2004; Michailidou et al., 2015, 2017; Shi et al., 2015, 2017; Lui et al., 2016). Also, injection of Aβ peptide in wild-type mice was shown to increase the levels of complement molecules, and in turn to promote synapse engulfment by microglia (Hong et al., 2016). A consistent role for complement in opsonizing synapses for removal has been shown in ageing models, with complement C3-deficient mice protected from age-related hippocampal decline (Shi et al., 2015). Complement upregulation was also reported upon viral infection. However, while such increase is critical for mediating microglial removal of presynaptic terminals in the hippocampus of West Nile Virus (WNV) infected mice (Vasek et al., 2016), it appears dispensable in IFN-γ mediated microglial synaptic stripping upon lymphocytic choriomeningitis viral infection (Di Liberto et al., 2018). Type I IFN signaling instead mediates synapse elimination by microglia in a mouse model of systemic lupus erythematosous (SLE), an incurable autoimmune disease (Bialas et al., 2017).

Purinergic signaling also plays crucial roles in microglia-mediated synapse refinement. ATP is a major signaling molecule, that acts as a danger signal once released extracellularly and profoundly affects microglial function (George et al., 2015; Rodrigues et al., 2015). Microglial processes are rapidly directed towards sources of ATP through the activation of P2Y12 receptors (Davalos et al., 2005). Activity-dependent synapse remodeling in the developing mouse visual cortex has been shown to strongly rely on such purinergic pathways in microglia, as knockout mice exhibited defective ocular dominance plasticity (Sipe et al., 2016). In addition, calcium-mediated purinergic receptors regulate microglial phagocytoses during postnatal brain development (Sunkaria et al., 2016).

Microglia not only sense and respond to ATP, but they can also serve as a source of purines, which modulate synaptic plasticity, thus representing an alternative mechanism for microglia-induced synaptic refinement (George et al., 2016).

Also, intrinsic glia dysfunction caused by genetic mutations can lead to aberrant synapse elimination. Loss of progranulin was shown to promote synaptic pruning by microglia, in a C1q-dependent manner (Lui et al., 2016). We have reported evidence for enhanced synapse engulfment, in the motor/somatosensory cortex of mice selectively lacking microglial TDP-43 (Paolicelli et al., 2017).

Other immune-related molecules such as CD47, have been recently described to work as “spare me” signals, and to protect synapses from excessive microglia-mediated pruning (Lehrman et al., 2018).

An interesting aspect that warrants further investigation is the cross-talk between microglia and astrocytes. Collecting evidence indicates that microglia can modulate astrocytic function, and conversely astrocytes can regulate microglial phenotypes (Jha et al., 2018). Microglial derived ATP, for instance, acts through the astrocytic receptor P2Y1, thus modulating excitatory neurotransmission and providing neuroprotection (Pascual et al., 2012; Shinozaki et al., 2014). Microglia can also induce neurotoxic astrocytes through the release of C1q, tumor necrosis factor-α (TNF-α), and interleukin-1α (IL-1α) (Liddelow et al., 2017). Recent findings show that astrocytic NF-kB activation induce a Wnt-dependent microglial proliferation, identifying astrocytes as important regulator of microglial expansion (Ouali Alami et al., 2018). Astrocyte-mediated synapse elimination has been shown to occur soon after acute sleep deprivation, before microglia-mediated remodeling, which is engaged only subsequently, if sleep deprivation is prolonged for several hours (Bellesi et al., 2017). It is thus tempting to speculate that astrocytes and microglia can act together in coordination, to ensure efficient synapse remodeling. Fine-tuned communication between microglia and astrocytes is crucial for proper brain functioning. Thus, a better understanding of the cellular processes involved in this cross-talk will be essential to elucidate the role of glia in the diseased brain.

Lifestyle factors related to dementia, such as nutrition, sleep quality and stress, are heavily implicated in glia-mediated synapse loss and cognitive decline (Cope et al., 2018; Rajendran and Paolicelli, 2018). On the other hand, large-scale human genetic studies have identified glia-specific genes as genetic risk factor in a range of neurological conditions from autism (Voineagu et al., 2011) to AD (Karch and Goate, 2015; Gosselin et al., 2017). Taken together, all this evidence reveals glia as a common link between many of the world’s most impenetrable diseases.

## Imbalance Between Excitation and Inhibition in Pathological States

The balance between the excitatory and inhibitory control of synaptic activity needs to be tightly maintained to ensure proper functioning and plasticity of neural circuits (Hensch and Fagiolini, 2005; Harauzov et al., 2010). Pre-synaptic terminals of excitatory synapses release glutamate as their major neurotransmitter, and are thus defined glutamatergic. Glutamate is received at the post-synapse by the ionotropic [N-methyl-D-aspartate receptors (NMDARs) and 2-amino-3-3-hydroxy-5-methyl-isoxazol-4-yl propanoic acid receptors (AMPARs)] and metabotropic (mGluR) glutamate receptors. On the other hand, pre-synaptic terminals at inhibitory synapses primarily release γ-aminobutyric acid (GABA), and are thus defined GABAergic. GABA is post-synaptically received by ionotropic (GABA_A_R) and metabotropic (GABA_B_R) GABA receptors. In terms of spatial organization, glutamatergic synapses are located almost exclusively on dendritic spines, whereas GABAergic synapses can be spread along the dendritic shaft, somata, and axon initial segments (Fritschy and Brünig, 2003; Penzes et al., 2011).

Excitatory neurons increase or decrease the accumulation of glutamate receptors at synaptic sites in response to changes in their own firing rates, through what has been defined as homeostatic synaptic scaling (Turrigiano et al., 1998; Turrigiano and Nelson, 2004; Turrigiano, 2008). The aim is to stabilize neuronal firing by adjusting its own synaptic strength to compensate for perturbations in surrounding neural activity (Ibata et al., 2008).

Network synchrony and oscillatory brain rhythms are promoted and controlled by the activity of inhibitory GABAergic interneurons across the entire lifespan, with important cross-talks with astrocytes modulating synaptic efficacy (Buzsáki et al., 2004; Klausberger and Somogyi, 2008; Perea et al., 2016; Sardinha et al., 2017; Mederos et al., 2018). Indeed, in the early stages of several brain disorders, impairment in inhibitory transmission combined with possible defects in homeostatic synaptic scaling at excitatory synapses might drastically compromise the excitation/ inhibition (E/I) balance (Palop and Mucke, 2016). Defective inhibition or aberrant excitation in brain development has been associated with severe alteration in the E/I ratio (observed in ASD for example) and considered to be causally linked to behavioral abnormalities (Rubenstein and Merzenich, 2003; Gao and Penzes, 2015; Nelson and Valakh, 2015). In support of this, modulation of prefrontal cortex E/I by optogenetics has recently been shown to rescue deficits in social behavior, in a mouse model of autism (Selimbeyoglu et al., 2017). Nevertheless, in other cases with memory alterations, selective changes of glutamatergic synaptic markers, have been reported without evident changes of GABAergic synaptic markers, in models of childhood epilepsy, diabetic encephalopathy and repeated stress, supporting the complexity of this topic (Cognato et al., 2010; Duarte et al., 2012; Canas et al., 2014; Kaster et al., 2015).

In neurodegeneration, such as in AD, network activities are altered even decades before clinical disease onset and are associated with diverse cognitive manifestations (Palop and Mucke, 2016). To date, the mechanisms and pathophysiological consequences of these alterations, which include activation and deactivation deficits of neural circuits, are poorly understood. Importantly, recent findings suggest that network activities can be experimentally or behaviorally manipulated to improve cognitive function in AD mouse models (Sanchez et al., 2012; Busche et al., 2015), and even in patients at risk of AD (Bakker et al., 2012, 2015). Overall, impairments in inhibitory connections, with consequent hyperexcitation, are emerging as potential mechanisms underlying cognitive dysfunction in several neurodegenerative diseases. On the other hand, selective increase in inhibition, *via* pharmacogenetic activation of parvalbumin interneurons, was recently shown to have beneficial effects, preventing stress-induced synapse loss *in vivo* (Chen et al., 2018).

Considering the complexity of our brain, it is easy to imagine that the fine-tuned balance between excitation and inhibition is not simply the net output of neuronal firing, but is rather the result of a highly regulated cross-talk amongst numerous cell types that are able to sense synaptic activity and to assist neurons to dynamically and appropriately adjust synapse strength and number. In this scenario, glial cells, such as astrocytes and microglia, which are known to closely interact with neural networks, can directly contribute to homeostatic synaptic scaling. Indeed, it has been proposed that glia participate directly in the homeostatic, activity-dependent regulation of synaptic connectivity through the release of TNF-α, a cytokine known to increase the cell surface expression of AMPA receptors (Stellwagen and Malenka, 2006). Similarly, several other molecules released by microglia and astrocytes might exert a direct regulation of plasticity by affecting receptor composition at the synapse, such as BDNF and IL1β (Parkhurst et al., 2013; Rizzo et al., 2018).

In addition to mechanisms mediated by release of soluble factors, it would be important to investigate whether refinement of synaptic connections by glial synaptic pruning also occurs to reinforce synaptic scaling. Growing evidence indicates that microglia are capable of sensing synaptic activity and act as key players in homeostatic regulation of neural firing (Li et al., 2012; Béchade et al., 2013; Ji et al., 2013). Lipopolysaccharide (LPS) injection in mice promotes transient but selective microglia-mediated removal of inhibitory synapses, which ultimately results in neuroprotection by suppressing inhibition and increasing synchronic neural firing (Chen et al., 2014). LPS-driven inflammation has profound effects on synaptic transmission (Pickering and O’Connor, 2007). Recent studies show that short-term LPS stimulation of microglia in spinal cord specifically decreases inhibitory glycinergic post-synaptic currents (Cantaut-Belarif et al., 2017). It is thus plausible to speculate that loss of excitatory synapses could be counteracted by microglia through removal of inhibitory inputs. On the other hand, astrocytes are much less motile, but their processes are more stably associated with synapses, and even considered a constant synaptic element, forming the so-called “tripartite synapse” (Araque et al., 1999). Interestingly, perturbations in astrocytic function lead to selective reduction in inhibitory, but not excitatory currents, as a consequence of rapid GABA depletion induced by downregulation of the glutamine synthetase enzyme (Ortinski et al., 2010).

Astrocytes also play critical roles in activity-dependent synapse elimination, as previously discussed. In the light of such observations, glia cells represent the perfect candidates for monitoring and eventually restoring E/I networks balance, through selective remodeling of excitatory or inhibitory inputs.

Overall, a dysregulated ratio between excitation and inhibition has significant implications in behavioral outputs associated with neurodevelopmental and neurodegenerative disorders, however the exact molecular and cellular processes at the origin of such dysfunction are yet to be elucidated. In the following paragraphs we will review the recent literature, highlighting possible links between glia-mediated synaptic remodeling and dysregulation of the E/I network balance in neurodegeneration.

## Alzheimer’s Disease

AD is the most common cause of dementia in the elderly, and despite its increasing prevalence there are no effective treatments available. The rare familial form of AD involves mutations of the amyloid precursor protein gene (APP) and Presenilins 1 and 2 (PSEN1 and PSEN2), which cleave APP to form Aβ species (Hardy and Higgins, 1992; Chávez-Gutiérrez et al., 2012). Late-onset (LOAD), or sporadic AD accounts for more than 95% of the Alzheimer’s cases, but has no clear etiology. With ageing being the strongest risk factor, several genetic polymorphisms in various gene loci have been associated with increased AD risk, such the Apolipoprotein E4 (ApoE4) allele or the R47H mutation in Trem2 (Roses, 1996; Guerreiro et al., 2013; Jonsson et al., 2013). AD is characterized by deposition of extracellular Aβ plaques, intracellular neurofibrillary tau tangles (NFTs), and progressive neurodegeneration accompanied by cognitive decline (Spires-Jones and Hyman, 2014). Several studies have focused on the pathological role of Aβ oligomeric species as a major player in neuronal and network dysfunction at early stages of the disease progression, thus providing a broad range of causative mechanisms (Cleary et al., 2005; Shankar et al., 2008; Li et al., 2011). For instance, Aβ can cause E/I imbalance through disruption of fast-spiking GABAergic inputs (Ren et al., 2018). Mutations in the APP leading to increase in Aβ oligomerization (E693 Osaka mutation; Tomiyama et al., 2010) have also been shown to cause selective GABAergic depletion in recessive familial AD (Umeda et al., 2017). Many of the LOAD risk genes, including APOE and TREM2, involve the brain’s immune system and the majority of them are highly enriched in microglia (Gosselin et al., 2017), suggesting glial cells are causally implicated in the pathogenesis of AD, and thus might be important players in the E/I imbalance observed already in the early stages (Henstridge et al., 2019).

**Figure 2:**
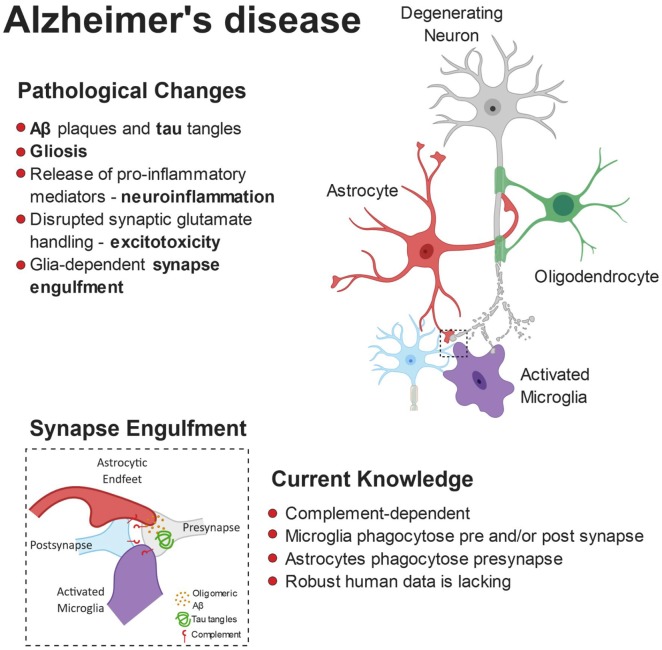
Pathophysiology of Alzheimer’s disease (AD). The build-up of pathological amyloid and tau species leads to neurodegeneration *via* numerous autonomous and non-autonomous pathways. Glial cells release pro-inflammatory mediators and lose their ability to regulate glutamate homeostasis, leading to synaptic dysfunction. Furthermore, the synaptic accumulation of proteins from the complement system leads to glial-dependent synapse engulfment and loss.

The microglial and astrocyte reactivity in AD and their physiological role in synaptic pruning has inspired a new wave of research into glial-mediated synapse loss in AD as a driver of network dysfunction (Serrano-Pozo et al., 2013; Rodriguez et al., 2014). Figure 2 summarizes the role of glia in mediating synaptic refinement in AD.

Other immune cells, such as lymphocytes and neutrophils may play important roles in the onset and progression of AD, also interacting with resident glia cells (Town et al., 2005; Xie and Yang, 2015; Ferretti et al., 2016).

### Microglia and Astrocytes in Synapse Loss in AD

It is well established that excitatory synapses are vulnerable in AD. Specifically, oligomeric Aβ (oAβ) not only induces synaptotoxicity but also synaptic weakening through prolonged long-term depression (LTD) and impaired LTP (Shankar et al., 2008; Li et al., 2009; Wu et al., 2010). In turn, glutamatergic signaling deficits in AD can range from NMDA and AMPAR internalization causing synaptic weakening (Zhang et al., 2003; Snyder et al., 2005) to NMDA-mediated glutamate excitotoxicity (Esposito et al., 2013). Recent reports suggest that other mechanisms of synapse dysfunction, such as the upregulation of adenosine A2A receptor might even occur before and independent of defective glutamate receptors, in a mouse model of AD (Viana da Silva et al., 2016).

Most studies demonstrating synapse loss by microglia have focused on the engulfment of pre- and post-synaptic markers, by co-localization approaches, with a preferential focus on excitatory synapses. The role of complement, in virtue of its role in mediating synaptic pruning during development, has been extensively investigated in the context of synapse elimination in AD. Indeed, in two amyloidopathy models of AD (Tg2576 and APP/PS1) crossed with C1q knockout mice, lack of C1q protected against synaptophysin loss in the hippocampus of aged mice (Fonseca et al., 2004). Similarly, more recent work has shown increased co-localization of C1q with excitatory post-synaptic densities-95 (PSD-95) in the J20 APP-overexpressing mouse model, as well as in transgenic APPsw/PSEN1DE9 mice, and also following injections of oAβ (Hong et al., 2016; Bie et al., 2019). Synaptotoxicity and LTP impairments induced by oAβ were also prevented in C1q knockout mice or upon administration of C1q neutralizing antibodies, suggesting C1q is critical for synaptic elimination (Hong et al., 2016; Bie et al., 2019). A proposed mechanism for C1q upregulation in hippocampal synapses is *via* metabotropic glutamate receptor signaling (mGluR1; Bie et al., 2019), which has been shown to be involved in synaptic LTD upon amyloid challenge (Chen et al., 2013). Overall, upregulation in complement molecules is associated with higher internalization of synaptic markers by microglia and with overall synaptic loss. In agreement with these outcomes, APP/PS1 mice lacking C3 showed a milder pro-inflammatory biochemical and morphological profile, reduced Aβ-associated microgliosis and astrogliosis, and greater levels of pre-synaptic (synaptophysin, VGLUT1) and excitatory post-synaptic (homer, PSD-95) markers compared to APP/PS1 mice expressing C3 normally (Shi et al., 2017). Importantly, C3 absence in 16-month-old APP/PS1 mice, spared cognitive deficits as shown by enhanced spatial memory. This suggests that in AD, healthy synapses that would not normally require physiological elimination may be aberrantly targeted by the complement system for elimination, partly eliciting the cognitive decline seen in AD.

Secretion of pro-inflammatory mediators by microglia is likely to occur concomitantly to phagocytosis, contributing to the AD-related synapse loss. Prolonged exposure to TNF-α in a triple transgenic AD-like model (3xTg) induced neuronal loss, microgliosis and upregulated C3 as well as intracellular Aβ levels (Janelsins et al., 2008). In the TgCRND8 AD mouse model, C3 was also upregulated in response to another potent pro-inflammatory cytokine, IFN-γ (Chakrabarty et al., 2010). Additionally, in culture assays, microglial IL-6 and nitric oxide (NO) have direct synaptotoxic effects on neurons (Azevedo et al., 2013). Therefore, microglia not only can directly mediate synapse elimination, but can also prime synapses for removal through released soluble factors.

The presence of the allele E4 for APOE (a major cholesterol carrier) is the strongest genetic risk factor influencing susceptibility to LOAD and it is associated with increased synapse loss (Koffie et al., 2009, 2012; Liu et al., 2013; Tzioras et al., 2018) as well as complement activation (McGeer et al., 1997). Transcriptomic studies have heavily implicated microglial APOE as a common facilitator of many AD-associated conditions, including amyloidosis, tauopathy, ageing and inflammation (Kang et al., 2018; Lin et al., 2018; Ulrich et al., 2018). Specifically, microglia close to Aβ plaques develop a disease associated phenotype and upregulate *Apoe* expression in a TREM2 dependent pathway (Keren-Shaul et al., 2017; Krasemann et al., 2017). APOE4 expressing mice also exhibit increased hippocampal gliosis and decreased levels of both synaptophysin and excitatory postsynaptic proteins (Zhu et al., 2012). Crossing APOE4 mice to the 5xFAD AD-like mouse model resulted in exacerbated Aβ-associated gliosis, presence of dystrophic neurites and IL-1β neuroinflammation (Rodriguez et al., 2014). Moreover, in human *post mortem* brains, the *APOE4* genotype is associated with an increase in microglial markers of activation including CD68, MSR-A and CD64 and decrease in homeostatic Iba1 (Minett et al., 2016). It is, therefore, compelling to hypothesize that, in carriers of the *APOE4* allele, microglia might be more prone to mediate pathological synapse loss.

Astrocytes and their many functions have been extensively studied in the context of AD (González-Reyes et al., 2017; Liddelow and Barres, 2017; Perez-Nievas and Serrano-Pozo, 2018), albeit their role in synapse loss is less clear. Both human and mouse studies have reported upregulation of reactive astrocyte signatures (GFAP) in the presence of an *APOE4* allele (Overmyer et al., 1999; Ophir et al., 2003; Belinson and Michaelson, 2009; Shi et al., 2017). In a recent study, induced pluripotent stem cells from APOE4 AD patients were differentiated into astrocytes and were then genetically modified using CRISPR-Cas9 to generate an *APOE3* genotype (Lin et al., 2018). This approach revealed that APOE4 to APOE3 conversion is sufficient to rescue the impaired phagocytic ability of astrocytes towards Aβ (Lin et al., 2018). Interestingly, and unexpectedly, an allele-dependent role for APOE was also shown in respect to mediating synapse elimination, with APOE2 enhancing and APOE4 decreasing the rate of synaptic pruning by astrocytes (Chung et al., 2016). This apparent controversy might be explained if we assume that homeostatic elimination of damaged synapses occurs constantly in the healthy brain. Thus, one could speculate that ApoE4 carrier would be impaired in such glia-mediated “homeostatic synapse remodeling.”

Only recently there was evidence of reactive astrocytes engulfing synapses in AD, with electron microscopy showing dystrophic VGLUT1-positive terminals being cleared by astrocytic endfeet in the hippocampus of APP/PS1 mice and in late stages of AD (Gomez-Arboledas et al., 2018). Whether this clearing mechanism is exacerbated in AD and contributes to excessive synaptic elimination is still under debate. The decreased phagocytic ability of reactive and APOE4-expressing astrocytes in development introduces new questions as to how these cells change in the context of AD and thus contribute to neurodegeneration.

### Glia Implications in E/I Imbalance in AD

Mounting evidence from mouse model studies suggest that, in the amyloid-depositing brain, functional impairments of local neuronal circuits lead to disruption in the E/I balance, which then result in large-scale networks defects (Busche et al., 2008; Busche and Konnerth, 2016; Palop and Mucke, 2016). Loss of inhibitory interneurons results in impaired oscillatory rhythm (Ramos et al., 2006; Baglietto-Vargas et al., 2010; Verret et al., 2012) leading to epileptiform activity (Vossel et al., 2016) and network hyperexcitability (Brown et al., 2018) in a subset of AD patients. Some studies have reported reduction of inhibitory pre-synaptic VGAT and GAT1 peri-somatic terminals on pyramidal neurons close to plaques, both in AD *post mortem* cases and aged APP/PS1 mice (Garcia-Marin et al., 2009). Others have found no such loss of inhibitory synapses in neither the same APP/PS1 model nor AD cases at comparable pathological stages; conversely, excitatory VGLUT1 boutons were found to be significantly reduced (Mitew et al., 2013; Canas et al., 2014). In the same study, Aβ was also suggested to increase astrocyte GABA synthesis, highlighting a possible implication of astrocytes as a source of E/I imbalance in AD (Mitew et al., 2013). Microglia, too, may play an active role in compromising the equilibrium of excitatory vs. inhibitory synaptic transmission in AD, by promoting loss of selective synapses (i.e., glutamatergic vs. GABAergic). Evidence for microglia engulfing excitatory inputs in AD mouse models have been provided (Hong et al., 2016; Paolicelli et al., 2017), however, evidence for the engulfment of inhibitory connections is still lacking. Whether microglia can directly contribute to E/I imbalance in AD is currently under debate, and further studies are required to investigate this possibility. A clear implication of microglia in the AD brain has been recently underscored by the use of PET tracers *in vivo*, which are capable of specifically revealing the microglial component of neuroinflammation (Edison et al., 2018; Horti et al., 2019).

## Parkinson’s Disease

PD is a neurodegenerative disorder characterized by massive degeneration of nigro-striatal dopaminergic neurons, which leads to progressive motor and cognitive symptoms. It is the second most common neurodegenerative disease and affects 2%–3% of the population over the age of 65 years (Poewe et al., 2017). The general term “parkinsonism” refers to the ensemble of movement disorders defined by the appearance of bradykinesia, rigidity or tremor. Cognitive impairment, in addition, is an important non-motor symptom of PD, with a mean duration from clinical disease onset to dementia of about 10 years (Aarsland et al., 2011; Selnes et al., 2017). A key neuropathological hallmark of the PD brain is the abnormal deposition of intraneuronal (Lewy bodies) and intraneuritic (Lewy neurites) fibrillary aggregates, mainly composed of α-synuclein (α-syn) and referred to as Lewy pathology. α-syn inclusions, initially thought to be limited to the substantia nigra pars compacta of the striatum, have been associated with the primary cause of neuronal loss in PD (Desplats et al., 2009). However, *post mortem* brain examinations of patients affected by PD revealed that Lewy pathology is not only confined to the striatum, but also affects other well-defined brain regions, possibly following a progressive spreading pattern (Del Tredici et al., 2002; Beach et al., 2009; Colom-Cadena et al., 2017). Staging of Lewy pathology in PD was first proposed by Braak et al. (2003), based on histological examinations showing the anatomical caudo-rostral progression of disease over time. Accumulating *in vitro* and *in vivo* evidence indicates that α-syn can undergo toxic conformational changes, spread from cell to cell, and initiate the formation of pathological aggregates, in a prion-like manner (Kordower et al., 2008; Li et al., 2008; Luk et al., 2012; Masuda-Suzukake et al., 2013). Together with the progressive stages of the disease, these data are in support of the spreading hypothesis, according to which Lewy pathology arises in specific brain nuclei and spreads to other structures through synaptic connections (Recasens and Dehay, 2014).

Transgenic animal models with α-syn overexpression exhibit neuronal dysfunction in the absence of cell loss, indicating that disruptions of synaptic transmission occur as an initial event, preceding α-syn-induced neuronal cell death (Janezic et al., 2013; Phan et al., 2017). Experimental evidence in fact shows that synaptic dysfunction is caused by presynaptic accumulation of α-syn aggregates, which impair axonal transport by affecting key proteins governing synaptic vesicle release (Kramer and Schulz-Schaeffer, 2007; Bellucci et al., 2012; Anichtchik et al., 2013).

Early synaptic dysfunction in PD has been supported by genetic evidence, with recently identified mutations in genes involved in clathrin-dependent synaptic vesicle endocytosis (SVE), such as DNAJC6 (auxilin) and SYNJ-1 (synaptojanin 1), in patients with juvenile and early-onset atypical parkinsonism (Nguyen and Krainc, 2018). In these models, a central role for glia cell have been also proposed, although causative mechanisms still await further supportive evidence (Teismann et al., 2003).

### Microglia and Astrocyte-Mediated Synapse Impairment in PD

Synapse loss in PD correlates with the pathological deposition of α-syn at the pre-synaptic site. Indeed, prolonged exposure to α-syn oligomers in hippocampal slices was shown to regulate synaptic transmission and impair LTP by activating NMDARs (Diógenes et al., 2012). Most of the studies aimed at elucidating the cellular basis of PD, have focused so far on mechanisms of neuronal dysfunction; however, PD-related genes are also expressed in astrocytes and microglia. Thus, it is likely that dysregulation of such genes may contribute to disease onset and progression *via* glia-mediated processes. Astrocytic roles in glucose metabolism are well described, and mutations in Parkin, PINK1, DJ-1 and LRKK2, associated with PD, have been shown to affect astrocytes function (Choi et al., 2013).

Parkin is a ubiquitin ligase largely implicated in PD, however its role in modulating glial specific function has just started to be unraveled. Recent studies show that parkin loss exacerbates inflammation and promotes survival of activated microglia by inhibiting necroptosis, thus contributing to chronic neuroinflammation (Dionísio et al., 2018), whereas in astrocytes it induces endoplasmic reticulum stress. Whether such effects can negatively impact on synaptic function and mediate synapse loss, however, remains to be elucidated. Similarly, novel evidence for DJ-1 modulation of glial function are emerging. DJ-1, encoded by *PARK7* gene, is a ubiquitously-expressed multifunctional protein which regulates anti-oxidant and anti-apoptotic gene expression (Canet-Avilés et al., 2004). DJ-1 knockdown in astrocytes was shown to impair astrocyte-mediated neuroprotection in primary neurons (Mullett and Hinkle, 2009; Kim et al., 2016), whereas astrocytic over-expression of DJ-1 prevented oxidative stress and mitochondrial dysfunction, leading to enhanced neuronal survival *in vitro* and *in vivo* (De Miranda et al., 2018; Frøyset et al., 2018). On the other hand, DJ-1 has been also shown to modulate microglial function, with its deficiency impairing autophagy, reducing α-syn phagocytosis and inducing a constitutive pro-inflammatory activation (Meiser et al., 2016; Nash et al., 2017). Mutations in LRKK2, another multifunctional protein associated with late-onset familial PD, has been shown to affect basic glial function. For instance, pathogenic mutations impair lysosomal function in astrocytes (Henry et al., 2015), and attenuate motility in microglia, preventing efficient response to brain damage (Choi et al., 2015). Altogether, these findings support the implication of glial dysfunction in the synaptic impairment occurring in PD.

### Glial Contribution to E/I Imbalance in PD

The pathophysiology of PD is characterized, among other features, by a prominent imbalance within striatal activity. Dopamine (DA) has excitatory effects on the projections from the striatum to the internal segment of globus pallidus (GP), defined as the direct pathway, acting through D1 receptors (D1Rs). The same neurotransmitter, however, exerts inhibitory effects on the projection from the striatum to the external segment of GP through D2Rs, or indirect pathway (Surmeier et al., 2007). Loss of DA, therefore, has complex consequences on multiple levels. Several studies in rodents, using both pathogenic 6-hydroxydopamine (6-OHDA) and 1-methyl-4-phenyl-1,2,3,6-tetrahydropyridine (MPTP) models, have shown that the progressive loss of striatal DA leads to a significant loss of glutamatergic synapses on medium spiny neurons (MSNs) of the dorsal striatum (Ingham et al., 1989; Zaja-Milatovic et al., 2005; Day et al., 2006), and that dendritic spines are decreased and enlarged specifically in the direct pathway neurons (Nishijima et al., 2014). Overall, the loss of dopaminergic input from the substantia nigra alters the equilibrium between excitatory and inhibitory control from the basal ganglia to the motor cortex.

Recent studies have highlighted the existence of subpopulations of astrocytes, with circuit-specific roles in the basal ganglia (Martín et al., 2015). Considering that both the direct and indirect pathways are fundamental for motor control, and are associated with motor deficits in PD and Huntington’s diseases, the selective regulation of specific synapses by astrocytes may be involved in the coordinated activity of these networks in the striatal function, therefore, pointing to astrocytes as central players in these disorders (Martín et al., 2015).

However, whether and how glia cells contribute to the E/I imbalance in PD remains elusive.

**Figure 3:**
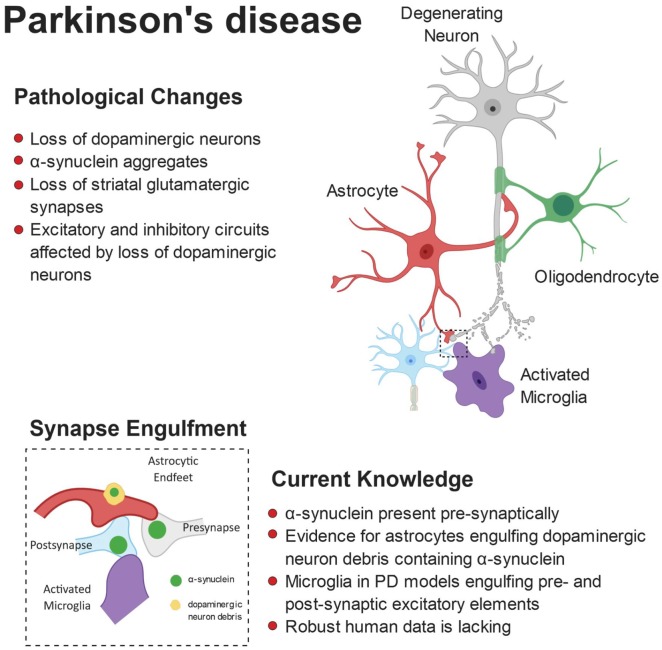
Pathophysiology of Parkinson’s disease (PD). Anatomically, PD is characterized by a loss of striatal dopaminergic neurons. This can lead to disruption of excitatory and inhibitory circuits, resulting in the clinical motor symptoms. Loss of glutamatergic synapses is apparent in the striatum and aggregates of α-synuclein (α-syn) are observed in the brains of patients. Furthermore, evidence suggests α-syn accumulates at the synapse, where both astrocytes and microglia have been shown to engulf α-syn-containing synaptic material.

It has recently been proposed that microglia may compensate for dopaminergic neuron loss through selective elimination of glutamatergic synapses from the subthalamic nucleus (Aono et al., 2017). By using the 6-OHDA-induced experimental Parkinsonism rat model, the authors showed a specific increase of activated microglia in the substantia nigra pars reticulata (SNr), engulfing excitatory pre- and post-synaptic elements. These findings suggest that microglia may be involved in a negative feedback in the indirect pathway of the basal ganglia to compensate for the loss of dopaminergic neurons in PD pathology (Aono et al., 2017). A central role for astrocytes has also been proposed in the PD brain, based on the observation that loss of DA neurons in the substantia nigra is associated with increased density of activated astrocytes (Hirsch et al., 2006; Gomide and Chadi, 2005; McGeer and McGeer, 2008). Only recently, however, it has been shown that striatal astrocytes engulf dopaminergic debris in the 6-OHDA model (Morales et al., 2017). Interestingly, α-syn was observed within astrocytic processes already 4 h after 6-OHDA administration, whereas the amyloid precursor protein (APP), found at synapses and accumulated in bulb-like structures of degenerating axons, was never found inside astrocytes. These findings suggest a selective engulfment of synaptic terminals by astrocytes, rather than a non-specific clearance of cellular debris (Morales et al., 2017). The contribution of microglia and astrocytes to synapse loss observed in PD is summarized in Figure 3.

## Amyotrophic Lateral Sclerosis (ALS)

ALS is caused by the breakdown of upper and lower motor neurons leading to the progressive weakness and atrophy of muscle, often resulting in respiratory failure and death within a few years of diagnosis. It is the most common form of MND, yet we still do not have a unifying theory of disease pathogenesis. Most cases (90%) are sporadic, with the remaining 10% due to known mutations in a growing number of disease-associated genes, such as c9orf72, SOD1, FUS and TDP-43 (Renton et al., 2014). Mounting evidence suggests that disconnection of the neuromuscular synapse occurs very early in the disease, with an initial toxic insult at the synapse, leading to disconnection of axons from their target cell, axonal breakdown and ultimately neuron death. This model led to the popular “dying back” hypothesis of disease progression (Frey et al., 2000; Fischer et al., 2004; Pun et al., 2006). This process has been described at both peripheral synapses at the NMJ and synapses in the CNS, however the toxic insult at either site has yet to be identified. An alternative theory is the “dying forward” hypothesis, which posits that breakdown of primary motor neurons in the brain leads to subsequent loss of secondary motor neurons in the periphery, and thus muscular denervation. Cortical hyperexcitability has been observed early in ALS brains using a number of imaging techniques and it is known that chronic hyperexcitability results in excitotoxicity, leading to motor neuron loss (Bae et al., 2013). In strong support of hyperexcitability as an important feature, the most widely prescribed drug for ALS, Riluzole, acts by dampening excitatory synaptic activity in the brain (Doble, 1996). Given the complex heterogeneity of ALS, it is likely that both dying forward and dying back processes occur in disease, however, regardless the nature of the predominant process, they ultimately converge on synaptic dysfunction. Cell autonomous and non-autonomous pathways have been studied in both pathogenic pathways, with glia strongly implicated in ALS progression.

### Synaptic Alterations in ALS

ALS has historically been considered exclusively a motor neuron disease, with much of the early research focused on the central and peripheral components of the motor system. Recent studies, however, have revealed that ALS is a multi-system disorder, displaying striking genetic, pathological and clinical overlap with frontotemporal dementia (FTD; Ling et al., 2013). Approximately 15% of ALS cases receive a co-morbid diagnosis of FTD and another 30%–40% present with milder cognitive and behavioral changes, reminiscent of symptoms (Strong et al., 2017). Given that synapse loss is the strongest correlate with cognitive decline in AD (Terry et al., 1991), it is interesting to note that synapse loss also associates with cognitive decline in ALS (Henstridge et al., 2018), suggesting that synapse loss may be a common feature of cognitive change in diverse neurodegenerative diseases (Henstridge et al., 2016).

Betz cells are giant pyramidal neurons located in layer V of the primary motor cortex where they project mono-synaptically onto lower motor neurons within the spinal cord. They receive synaptic input primarily from the premotor cortex, which is important for the planning and execution of complex movement. Research has shown that synapses onto anatomically-normal Betz cells are dysmorphic in the brains of ALS patients (Sasaki and Iwata, 1999). Furthermore, diverse animal models of ALS have revealed a common feature of pre-symptomatic loss of cortical synapses (Qiu et al., 2014; Fogarty et al., 2015, 2016). Lower motor neurons within the spinal cord exhibit a lower density of axo-somatic synapses in ALS (Sasaki and Maruyama, 1994a, b), suggesting a disconnection between upper and lower motor neurons. At the periphery, loss of NMJ synapses represent one of the first anatomical changes in ALS models, occurring long before disease symptoms (Frey et al., 2000; Fischer et al., 2004; Pun et al., 2006). Collectively, these studies show that synaptic connections throughout the motor system are vulnerable early in disease.

#### Microglia-Dependent Loss of Central Synapses in ALS

Mutations in superoxide dismutase 1 (SOD1), an antioxidizing enzyme, are associated with ALS.

Animal models overexpressing human mutated SOD1, display pre-symptomatic changes to cortical motor neurons, resulting in intrinsic hyperexcitability (Saba et al., 2016) and an early loss of inhibitory interneurons (Clark et al., 2017). Thus, it appears in SOD1 animal models of ALS, both intrinsic hyperexcitability and decreased inhibitory control play a role in cortical pathophysiology. Recent human studies using novel neurophysiological techniques have also suggested that imbalance between intracortical excitatory and inhibitory systems leads to hyperexcitability (Van den Bos et al., 2018). Many diverse ALS models exhibit pre-symptomatic synapse loss. SOD1 models present with early spine loss in the motor cortex, which worsens with disease progression (Fogarty et al., 2015; Saba et al., 2016). Mice overexpressing mutated forms of two RNA-DNA binding proteins commonly associated with ALS display overt synaptic defects: TDP-43 A315T mice have progressive loss of spines from P60–P90 compared to wild type mice (Handley et al., 2017) and FUS R521G mice have a significantly lower density of mature spines in the cortex at P18 (Sephton et al., 2014). While cell autonomous changes can influence neuronal morphology, glial cells also have the ability to significantly influence both excitatory and inhibitory synaptic systems *via* the release of toxic mediators or by direct phagocytosis of neuronal compartments, as described above. The study of microgliosis in human ALS tissue has mostly been confined to *post mortem* studies, which tend to show an increase in microglia number (Kawamata et al., 1992; Brettschneider et al., 2012). However, some studies have shown increased microglial activity in live human ALS brain, using PET imaging (Turner et al., 2004; Zürcher et al., 2015). Microglial activation is consistently detected in the motor cortex however one study also detected an increase in the dorsolateral prefrontal cortex and thalamus (Turner et al., 2004). Interestingly, microgliosis appears to associate with disease severity (Turner et al., 2004; Brettschneider et al., 2012; Zürcher et al., 2015), with a recent study suggesting microgliosis is specifically associated with rapid disease progression (Gorter et al., 2018). Taking a data-driven approach, another recent work uncovered networks of genes that associate with motor neuron pathology in human ALS brain. The study found that most genes within the top scoring network, are expressed in microglia (Cooper-Knock et al., 2017). Furthermore, TREM2 levels in the cerebrospinal fluid (CSF) of ALS patients was higher than controls and TREM2 levels positively correlated with disease duration in late stage ALS (Cooper-Knock et al., 2017). This supports previous work which found an increased expression of TREM2 mRNA in human and SOD1 mouse spinal cord, and also implicated a rare variant in TREM2 (p.R47H) as a risk factor for developing ALS (Cady et al., 2014). Therefore, it is clear that microglia have an important role to play in ALS pathogenesis (Geloso et al., 2017), but what effect are microglia having on surrounding neurons? An intricate mouse study utilizing cell-type specific expression of mutant SOD1 G93A, placed microglia in a central role for mediating ALS progression. Removing mutant SOD1 from microglia, thus returning them to a wild-type state, had no effect on disease onset, but significantly slowed late stage progression (Boillée et al., 2006). This is supported by recent work (Frakes et al., 2014) showing that a toxic microglial gain of function exerts a pathological effect on neurons in ALS. Our recent work has uncovered a potential mechanism by which activated, inflammatory microglia may exert degenerative effects on neurons. TDP-43 is the main pathological hallmark of ALS, with protein aggregates found in almost 100% of ALS cases (Neumann et al., 2006; Ling et al., 2013). Debate surrounds whether this leads to a pathological loss of normal function or a toxic gain of function, however, when TDP-43 is specifically knocked-out of microglial cells in mice, we found that they convert to a hyper-phagocytic phenotype and ingest surrounding synapses (Paolicelli et al., 2017). This links TDP-43 pathology to microglial activation and synapse loss. In human ALS brain, the presence of TDP-43 pathology in the frontal cortex is associated with a higher burden of microglial activity as evidenced by increased CD68 expression (Brettschneider et al., 2012; Paolicelli et al., 2017). Furthermore, the presence of TDP-43 in the frontal cortex was associated with lower synapse number in one study (Henstridge et al., 2018) and cognitive impairment in another (Brettschneider et al., 2012). Taken together, these studies place TDP-43 pathology and activated microglia at the sites of synapse loss in the ALS brain, resulting in a breakdown of neuronal function and clinical manifestation of ALS.

The evidence above clearly states that synapse loss is a prominent feature in human ALS brain and in diverse ALS models. However, does a similar synaptic breakdown occur around lower motor neurons within the spinal cord? A number of early studies assessing synaptic coverage of spinal motor neurons in human *post mortem* tissue described synapse loss and altered morphology in remaining synapses (Matsumoto et al., 1994; Sasaki and Maruyama, 1994a, b; Ince et al., 1995). Similar findings are evident in the SOD1 G93A mouse model, with decreased synapses onto motor neurons in the spinal cord and decreased spine density of spinal motor neurons (Zang et al., 2005; Fogarty et al., 2017). In the same mouse model, another study found a decrease in total synapse number onto brainstem motor neurons that manifested as a small increase in excitatory synapses and a larger decrease of inhibitory terminals (Sunico et al., 2011). Taken together it is clear that synapses are lost in the brainstem and spinal cord in ALS. At approximately the same time as spines are being lost in the SOD1 G93A mouse model, microglia are proliferating in the rat SOD1 G93A model (Graber et al., 2010). However, to the best of our knowledge no studies to date have assessed whether microglia may be stripping synapses in the spinal cord.

A recent study has found that microglia in the spinal cord may play a neuroprotective role. When human TDP-43 was over-expressed exclusively in neurons, microgliosis in the spinal cord was mild, but when the TDP-43 was switched off with doxycycline treatment, microglia became inflamed, proliferated and selectively engulfed neuronally-derived TDP-43 (Spiller et al., 2018). This has been recently confirmed in a zebrafish model of human TDP-43 over-expression, in which microglia actively phagocytose degenerating spinal cord neurons expressing TDP-43 (Svahn et al., 2018). Taken together, these studies suggest that TDP-43 in stressed neurons may act as a signal to attract phagocytic microglia to clear away aggregated TDP-43. With this in mind, it is interesting to note that TDP-43 aggregates have been observed in human synapses (Henstridge et al., 2018) and may act as a microglial “eat me” signal in the same way complement appears to in AD.

Microglia can also exert indirect effects on neuronal and synaptic function by the release of numerous signaling molecules. There is a wealth of literature describing the increased expression of proinflammatory mediators in ALS models and patients, ranging from elevated blood levels of TNF-a in human blood to increased chemokine MCP-1 expression in SOD1 mouse models (reviewed in Philips and Robberecht, 2011). Many of these excreted molecules can directly affect neuronal physiology, such as NO, reactive oxygen species (ROS) and cytokines (Henkel et al., 2009), further supporting a role for microglia ALS-related synapse dysfunction.

#### Astrocyte-Dependent Loss of Central Synapses in ALS

Glutamate is the major excitatory neurotransmitter in the brain and its levels need to be tightly controlled at the synapse to prevent excitotoxicity. Astrocytes play a major role by actively taking up excess glutamate using glutamate transporters, EEAT1 and EEAT2 (also known as GLAST and GLT-1, respectively). In SOD1 models, GLT-1 levels decrease as disease progresses (Bruijn et al., 1997) and this finding is consistent in human ALS spinal cord and brain (Rothstein et al., 1995). These early studies suggest that a failure in astrocytic control of synaptic glutamate may result in excitotoxicity and network imbalance, supported by a study that knocked out glial GLT-1 using oligonucleotides and discovered that animals developed a progressive motor paralysis (Rothstein et al., 1996). Interestingly, crossing the SOD G93A mouse with an EAAT2 over-expressing mouse delayed axonal dystrophy and motor neuron loss but did not affect onset of paralysis or life span (Guo et al., 2003). Despite this less than positive outcome, a pharmacological approach (beta-lactam antibiotic, ceftriaxone) to stimulate GLT-1 expression in SOD1 G93A mice at symptom onset, led to delayed loss of muscle strength and body weight and prolonged life by 10 days (Rothstein et al., 2005). Ceftriaxone was tested in a recent clinical trial and provided some excitement after a successful Phase 2, however it failed to show clinical efficacy in Phase 3 (Cudkowicz et al., 2014). It was not determined if the drug affected EAAT2 expression or function in the participants, so further work is required to assess the value in targeting glial glutamate transporters in ALS. Astrocytes also play an important trophic role through the uptake of glucose from the blood stream, which they convert into lactate and pass to neurons for the generation of glutamate (Pellerin and Magistretti, 1994; Pellerin et al., 1998). Lactate is shuttled from the astrocyte to the neuron in a pathway requiring the glutamate transporters mentioned above, however pre-symptomatic SOD1 G93A mice have a significantly lower amount of lactate in spinal cord homogenates and a decreased expression of GLAST (Ferraiuolo et al., 2011). This suggests a disruption in the astrocyte-neuron lactate shuttle, potentially rendering the neurons hypometabolic.

Small heat shock proteins (HSPBs) are important chaperones that reduce protein misfolding and aid in misfolded protein degradation. A recent study has found that in human ALS spinal cord, rapidly progressing disease was associated with increased HSPB5 and HSPB8 in astrocytes (Gorter et al., 2018). Furthermore, a recent rat model with restricted mutant human TDP-43 (M337V) expression in astrocytes, displayed a progressive paralysis due to loss of motor neurons in the spinal cord (Tong et al., 2013). This strongly supports an important role for glia-derived toxicity in ALS. These studies suggest that astrocytes may become overwhelmed with misfolded protein stressors in ALS, which could affect their trophic support of neurons and synapses. While it is clear that astrocytes have an important role to play in the synaptic pathology of ALS, there are currently no studies that we are aware of showing astrocytic ingestion of synaptic terminals. It will be important to discover if astrocytes are restricted to indirect effects on synaptic dysfunction or whether they can physically strip synapses and dystrophic dendrites as observed in other diseases.

**Figure 4:**
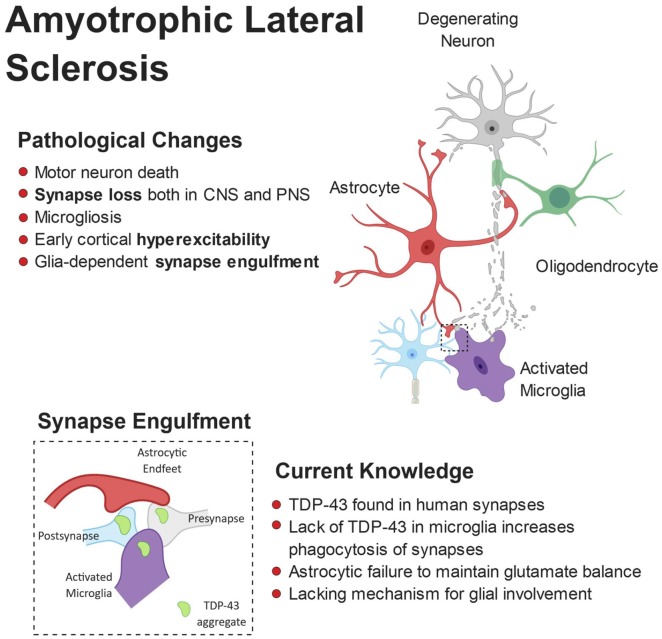
Pathophysiology of amyotrophic lateral sclerosis (ALS). ALS is characterized by the breakdown of motor neurons in the motor cortex and spinal cord. Gliosis and cortical hyperexcitability are early features of the ALS brain and aggregates of TDP-43 are found in almost all patients. TDP-43 has been found at human synapses in ALS and the removal of TDP-43 from microglia leads to hyperphagocytic cells that engulf synapses. Microglia have been shown to engulf neuronally-derived TDP43. Interestingly, synapse loss is an early feature of ALS and observed in both the central and peripheral nervous systems.

Cross-talk between astrocytes and microglia also appear to play a critical role in ALS pathogenesis. In the SOD1 G93A mouse, specific knock out of SOD1 G93A from astrocytes, thus reverting them back to wild-type, had no effect on ALS onset but significantly delayed microglial activation and slowed late-stage disease (Yamanaka et al., 2008). This suggests that in ALS not only do microglia and astrocytes affect neuronal physiology alone, they also regulate the function of one another. Figure 4 shows a schematic representation of microglial and astrocytic roles in synapse loss in ALS.

### Glia-Dependent Loss of Peripheral Synapses in ALS

The NMJ exists as a tripartite structure, consisting of the motor nerve ending, the postsynaptic muscle cell and non-myelinating perisynaptic Schwann cells (PSCs; Ko and Robitaille, 2015). These specialized glial cells are critical for the maintenance and remodeling of adult NMJs, actively phagocytosing damaged nerve terminals and guiding regenerating nerves to their correct target (Ko and Robitaille, 2015). Active uptake of degenerating axonal components by Schwann cells involves the initial formation of “axosomes,” aggregates of synaptic proteins and membrane fragments that are released by the axonal tip (Bishop et al., 2004). Phagocytic behavior of PSCs is induced by signals released from degenerating motor neuron axons, resulting in engulfment of synaptic terminals at the NMJ (Duregotti et al., 2015). Interestingly, in a toxin-induced neuropathy model, the toxic signals (H_2_O_2_, mitochondrial DNA and cytochrome C) are released from mitochondria within the degenerating motor nerves, supporting the role of mitochondrial dysfunction in ALS (Duregotti et al., 2015; Smith et al., 2017). Furthermore, the expression of numerous receptors and signaling molecules involved in regulating PSC activity is under the control of the RNA-binding protein TDP-43 (Narayanan et al., 2013), suggesting that TDP-43 dysfunction can significantly impact the activity of Schwann cells at the NMJ.

Given the important role of the complement system in synapse loss in AD, it is interesting to note that components of the complement system are found at the NMJ in SOD1 mouse models and human tissue (Heurich et al., 2011; Bahia El Idrissi et al., 2016). It will be interesting to discover if these proteins tag the synaptic terminals for engulfment, in a similar glial-dependent process as described in AD above. Paradoxically, a recent study has shown that C1q deletion exacerbates disease progression and synapse loss in a SOD1 mouse model, revealing that further study is required to understand the role of complement at peripheral synaptic function (Lobsiger et al., 2013).

While these intriguing studies provide a glimpse of the normal function of PSCs, little is known about their role in disease. For example, it would be important to know if disease-associated changes in PSC activity resulted in aberrant synapse loss or whether their trophic role is disrupted in disease, leading to pathogenic processes.

## Multiple Sclerosis

**Figure 5:**
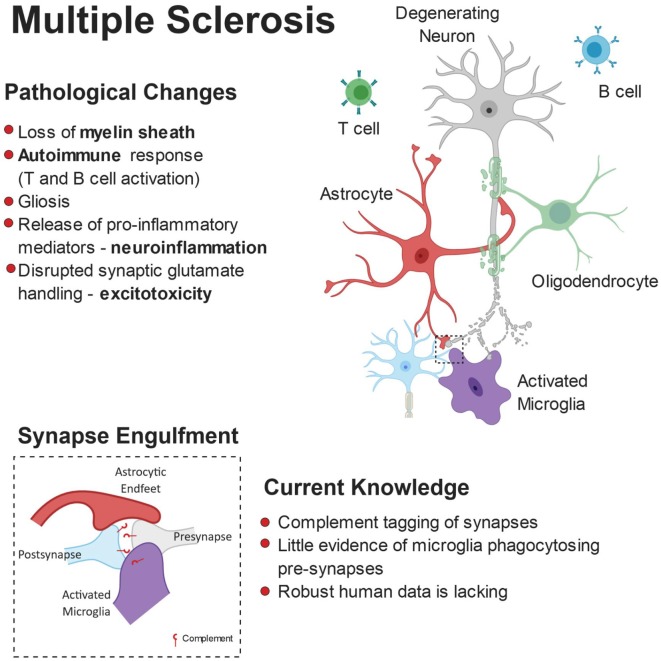
Pathophysiology of multiple sclerosis (MS). While myelin loss is a central feature of MS pathology, it is accompanied by neurodegeneration, gliosis and immune cell (B-cells and T-cells) infiltration. Release of pro-inflammatory mediators and disrupted glutamate handling by glial cells leads to a toxic neuronal milieu. Furthermore, there is evidence that microglia are involved in complement-dependent synapse engulfment.

MS is an autoimmune disease characterized by oligodendroglial dysfunction (Kotter et al., 2006; Miron et al., 2013) and T-cell driven inflammation (Korn et al., 2007; Aggelakopoulou et al., 2016), resulting in demyelination of gray and white matter tracts. Loss of the myelin sheath makes axons less capable of propagating electrical signals to the synapse and renders them more vulnerable to degeneration. Typically, affected individuals present with motor deficits but signs of cognitive decline are also evident in some patients (Rao et al., 1991; Chiaravalloti and DeLuca, 2008). Due to the demyelinating nature of MS and because the white matter is myelin-rich, changes in the gray matter have been largely over-looked, particularly in respect to synapses. Dendritic cortical spine loss, independent of cortical demyelination and axon loss, has emerged as a pathological feature of some MS patients (Nistico et al., 2014; Jürgens et al., 2016), which may explain the cognitive deficits (Di Filippo et al., 2018). The role of microglia and astrocytes as active players in MS-associated synaptic stripping (Figure 5) has been implied by multiple *post-mortem* studies but quantitative and mechanistic evidence is still elusive.

### Microglial and Astrocyte Contribution to Synapse Loss in MS

Hippocampal microgliosis is a common feature in MS models as well as in human *post-mortem* samples, highlighting microglia yet again as a potential driver of synaptic loss in disease. Primarily, there are fewer pre-synaptic terminals in demyelinated MS cases (MS-D) compared to myelinated (MS-M) and control cases in various regions of the hippocampus, including CA3 and CA1 (Michailidou et al., 2015). Moreover, the researchers found that the levels of C1q and C3 are increased in the MS-D cases, and have shown, but not quantified, activated microglia containing pre-synaptic elements, suggesting that complement molecules—once again—may act as a synapse removal tag. C3d-expressing microglial clusters are seen in chronic MS lesions rather than the acute phases of demyelination, indicating C3d may not play a role in the initial synaptic degeneration seen in MS-D. Moreover, in the gray matter of MS *post-mortem* cases, C1q-positive neurons show dysmorphic nuclei, typical of cell stress, when adjacent to activated microglia clusters (Watkins et al., 2016). Together, these studies suggest a model in which C1q may act as a tag for early synaptic engulfment in MS, while neuronal C3d is internalized by microglia during phagocytosis of degenerating neuronal and synaptic debris in later phases of the disease. In addition, other members of the classical complement cascade need to be considered. Specifically, administration of oligonucleotides against C6 partially rescued the synaptophysin depletion found in experimental autoimmune encephalomyelitis (EAE) mouse model for MS. Reduction in C6 also led to decreases in the levels of IL-1β, microgliosis, myelin damage, and C9 of the membrane attack complex (MAC; Michailidou et al., 2018). Interestingly, C9 showed a strong negative correlation to synaptophysin, meaning high levels of C9 correlate with lower levels of pre-synaptic terminals. Given that the MAC can activate subsequent pathological mediators like the NLRP3 inflammasome in microglia (Laudisi et al., 2013), which allows maturation and release of IL-1β (Jo et al., 2016), it makes sense that there are lowers levels of inflammation and gliosis when C6 is inhibited. Furthermore, it has been previously discussed in the context of AD that other pro-inflammatory cytokines secreted by microglia have synaptotoxic effects, providing an alternative pathway to non-contact dependent synapse loss. Astrocytes can also contribute to glutamate excitotoxicity in MS as they reduce the levels of their glutamate transporters, EAAT1 and EAAT2, in MS-D lesions (Dutta et al., 2011), allowing excess levels of glutamate to surround synapses.

### E/I Imbalance in MS and Possible Implication of Glia

Evidence of E/I imbalance has been reported in MS animal models, particularly the EAE model. Electrophysiological experiments have shown decreased excitatory post-synaptic potentials (EPSPs) in the CA1 of the hippocampus and impaired LTP in EAE mice, leading to cognitive impairments mediated by IL-1β driven inflammation (Kim et al., 2012; Di Filippo et al., 2013). This functional impairment may arise due to the downregulation of GluN2B NMDAR subunits in EAE mice. In contrast, there is evidence for inflammation-associated increase of LTP and reduction of LTD in EAE mice, displaying overall circuit hyperexcitation (Nistico et al., 2014). In favor of this, a magnetic resonance spectroscopy study found increased levels of glutamate in demyelinated brain areas of MS patients (Srinivasan et al., 2005), implicating excitatory imbalance as a pathological substrate for myelin damage, preceding synapse loss (Dutta et al., 2011). Specifically, oligodendroglia are vulnerable to glutamate excitotoxicity as they express NMDARs (Pérez-Otaño et al., 2016) which are required for activity-dependent myelination and plasticity (Lundgaard et al., 2013). Therefore, initial hyperexcitability could result in oligodendroglial dysfunction and demyelination, ultimately rendering synapses weaker and more vulnerable to elimination, leading to later LTP impairments.

However, other studies have reported synapse reduction occurring independently of demyelination (Jürgens et al., 2016; Albert et al., 2017).

Researchers also found increased, rather than decreased, spine density in the somatosensory cortex of EAE mice, associated with increased VGLUT1 levels and disrupted PV+ interneuron connectivity (Potter et al., 2016). The altered excitatory-inhibitory balance in the cortex of these mice was associated with increased density of Iba1+ microglia, however no evidence of cause-effect was reported (Potter et al., 2016).

The synaptic terminals assessed in the above MS and EAE studies are exclusively pre-synaptic with no distinction of excitatory or inhibitory nature. Loss of inhibitory signaling causes E/I imbalance, which has already been described here in the context of dementias but applies to MS as well. Indeed, GABA levels are reduced in the CSF of patients with MS, indicating decreased inhibition (Manyam et al., 1980). More recently, RNA sequencing from gray matter of motor cortices in MS patients showed downregulation of multiple genes that are critical to interneuron function (Dutta et al., 2006). Namely, there was downregulation of GAD67, an enzyme required for GABA synthesis pre-synaptically, and of the GABA receptor subunits α1 and β3 which are essential for GABA function post-synaptically. Furthermore, parvalbumin (PV) and cholecystokinin (CCK) levels were found to be lower in MS than controls, with PV-positive interneurons reduced by 30% in MS gray matter (Dutta et al., 2006).

Reduction of axosomatic synaptic terminals was recently reported in the cerebellum of MS patients, associated with increased levels of reactive astrocytes and microglia, specifically in the dentate nucleus (Albert et al., 2017). In this study, ultrastructural examination by electron microscopy revealed evidence for astrocyte-mediated synaptic stripping (Albert et al., 2017).

Altogether, these findings point toward a consistent alteration in the E/I balance in MS and encourage further investigation to better elucidating the role of glia mediated-synapse loss.

## Conclusion

**Figure 6:**
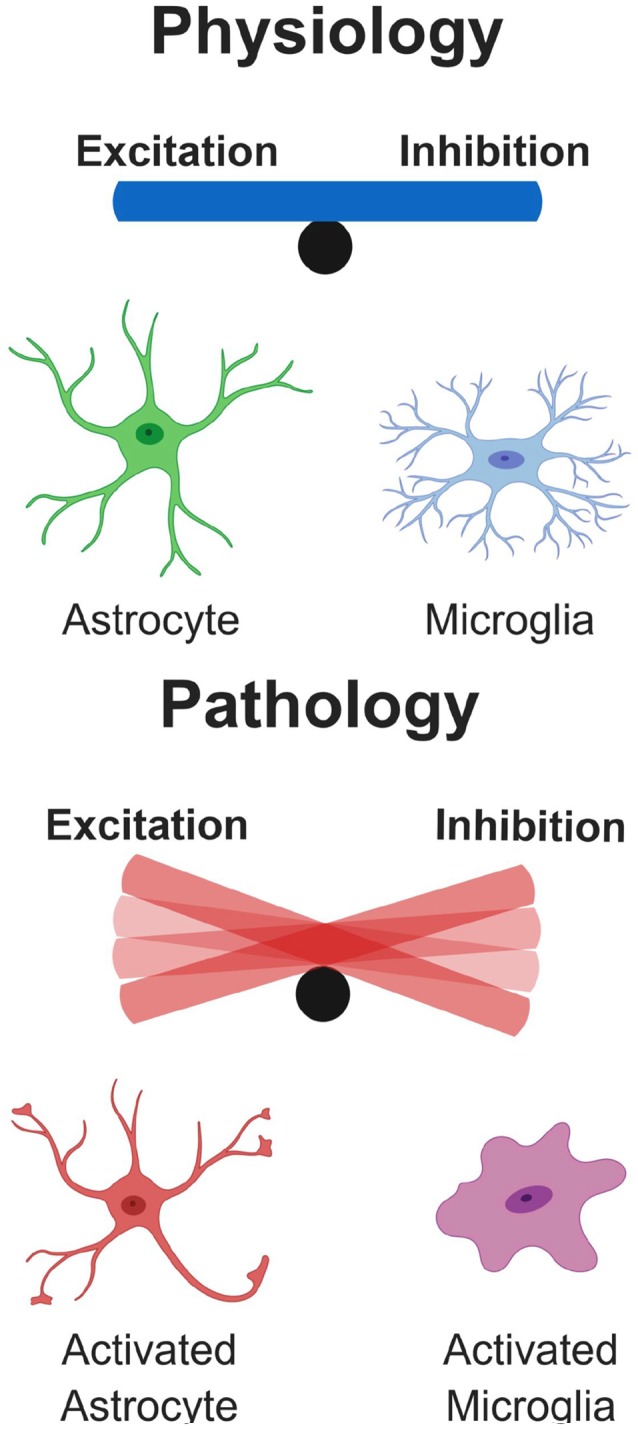
Glial Influence on excitatory/inhibitory balance. Under physiological conditions, glial cells play important roles in the control of neuronal physiology, resulting in a well-controlled balance of excitatory/inhibitory neuronal networks. However, under pathological conditions as described in some of the diseases here, glial cells become hyperactive and damage surrounding neurons. This results in a dramatic tip of the balance depending on whether excitatory or inhibitory cells are disproportionally affected in the network.

Here, we have summarized the contributions of glial cells in some of the most common neurodegenerative diseases, highlighting evidence for their role in synapse remodeling. In disease, glia-mediated synaptic refinement likely represents an attempt to counteract network dysfunction occurring in the early stages of the disease. In this scenario, glia selectively remove excitatory or inhibitory connections in specific brain regions, to compensate for disease-associated changes in synaptic input. On the other hand, intrinsic dysfunction of glia cells, due for instance to genetic mutations, could also play a critical causative role in the pathogenesis of the diseases, acting as a primary trigger for E/I imbalance, by inducing excessive synaptic pruning (Figure 6). It is tempting to speculate that similar mechanisms could occur in response to shifts in E/I balance in the developing brain, where glia-mediated synaptic alterations may lead to long lasting structural and functional defects, thus promoting the risk of developing psychiatric disorders and depression later in life (Durieux et al., 2015; Rial et al., 2015). Thus, deeper insight into the process of synapse remodeling mediated by glia cells, both in physiological and pathological conditions, will be essential for designing effective therapeutics to prevent, or at least halt synapse elimination. Such therapeutic interventions include an attenuation of the microglial response in AD pathology. For example, in two separate models of AD, the APPswe/PS1 and the 5xFAD, inhibiting the colony stimulating factor-1 receptor (CSF1R) markedly reduced microglial proliferation, rescuing synapse loss and cognitive deficits (Olmos-Alonso et al., 2016; Spangenberg et al., 2016). Importantly, in neither of these studies did pharmacological inhibition of microglia influence Aβ-plaque load, suggesting that microglial activation in AD can be synaptotoxic, *via* non-Aβ mediated mechanisms. However, whether this synaptic and cognitive rescue was due to attenuating microglial driven inflammation is unclear. Microglial neurotoxic and synaptotoxic cytokine release in prodromal stages of the disease is likely to coincide with complement deposition and aberrant phagocytosis. Complement molecules deposited at synapses have been reported to be work as a powerful “eat me” signal in several distinct neurodegenerative disorders. However, synapse elimination in pathological contexts could also be seen as a beneficial process, at least in the initial stages, aimed at re-establishing the E/I balance. Critical information will be provided by clinical trials currently testing a humanized anti-C1q antibody in neurodegeneration, after its safety was recently proven in both animal models and human cohorts (Lansita et al., 2017).

Given that early synapse loss is a common phenotype in many neurodegenerative diseases, it raises the exciting possibility that a greater understanding of glia-mediated synapse loss may lead to a single therapeutic strategy that targets many of the world’s most devastating diseases.

## Author Contributions

CH, MT and RP revised the literature and wrote the manuscript.

## Conflict of Interest Statement

The authors declare that the research was conducted in the absence of any commercial or financial relationships that could be construed as a potential conflict of interest.
